# Supplementary material for: A New Mechanism in THRA Resistance: The First Disease-Associated Variant Leading to an Increased Inhibitory Function of THRA2
Source: Int J Mol Sci. 2021 May 19;22(10):5338. doi: 10.3390/ijms22105338 (PMC8159125; doi:10.3390/ijms22105338)
Supplement: Supplementary file 1 [file ijms-22-05338-s001.zip › ijms-1156618-supplementary.pdf]

# A new mechanism in THRA resistance: the first disease-associated variant leading to an increased inhibitory function of THRA2

*Sarah Paisdzior, Ellen Knierim, Gunnar Kleinau, Heike Biebermann, Heiko Krude, Rachel Straussberg, Markus Schuelke*

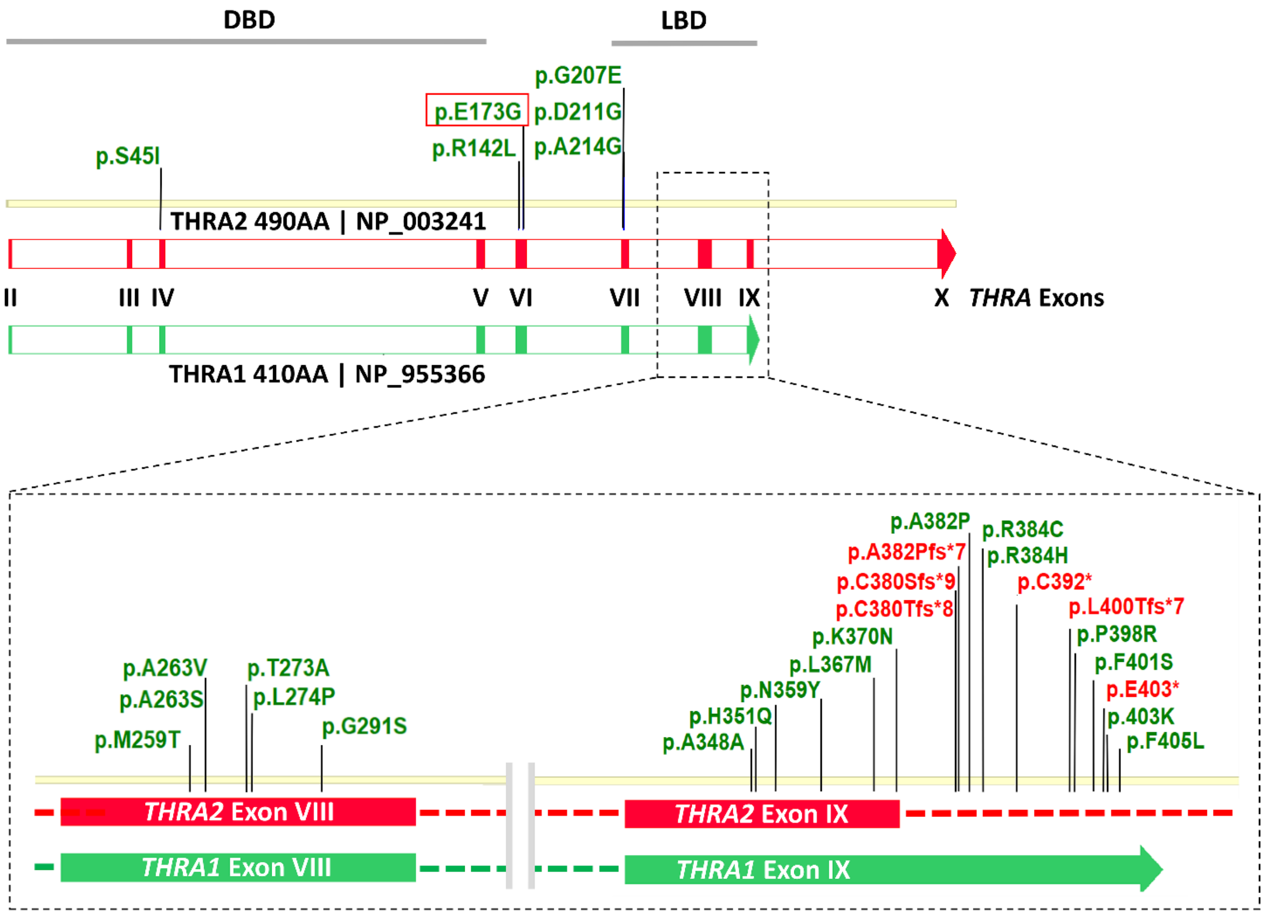


**Figure S1**: The two main alternative splicing isoforms of the *THRA* gene. The protein coding exons II-X are depicted between the splicing isoforms. Exon I is non-coding. The T3 binding pockets are encoded mainly by exon 9 of the THRA1 isoform (green). Most of the reported disease-associated genetic variants are located in the ligand (T3) binding domain (LBD). The DNA-binding domain (DBD) of the receptor is located at the N-terminus of the protein. The here described disease-associated variant p.E173G is located between DBD and LBD (red square). AA, amino acid. Missense variants are highlighted in green, frameshift and nonsense variants are highlighted in red.

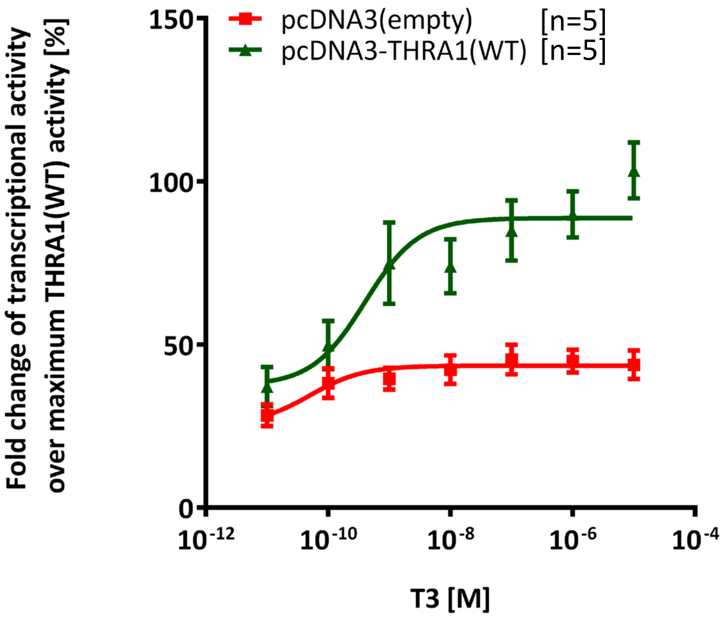


**Figure S2**: Results from concentration-response experiments of transiently transfected JEG3 cells with either pcDNA3-THRA1(WT) or empty pcDNA3 vector as mock control. Even mock transfected cells did respond to T3, although effects are small in comparison to the transfection with the THRA1(WT) construct. Values from the luciferase assay were normalized to the maximum transcriptional activation of THRA1(WT) [=100%]. The dots and the error bar depict the mean and SEM of five independent experiments performed in triplicates

**Table S1**: **Disease associated variants in *THRA* that have been published to date**. In case *in vitro* functional tests have been performed using the recombinant protein with the respective variant inserted (e.g. *via* T3 stimulated transactivation by THRA1 in a reporter gene assay), we mention, whether the authors had found a transactivation defect that could be (partially) overcome by rising T3 concentrations. *, variant was only published in the ClinVar database (URL https://www.ncbi.nlm.nih.gov/clinvar/?term=THRA[gene]) and no functional tests have been performed. The ClinVar accession number is provided. n.i., no information available. Missense variants are highlighted in green, frameshift and nonsense variants are highlighted in red.

| **THRA1 isoform** | | **THRA2 isoform** | | **T3 stimulated transactivation by THRA1** | **Genomic location** | **Publication** |
|---|---|---|---|---|---|---|
| **NM_199334** | **NP_955366** | **NM_003250** | **NP_003241** | | **GRCh37.p13 (hg19)** | |
| c.134G>T | p.(S45I) | c.134G>T | p.(S45I) | n.i. | 17:38233773G>T | CinVar VCV000029913* |
| c.425G>T | p.(R142L) | c.425G>T | p.(R142L) | n.i. | 17:38240917G>T | CinVar VCV000803389* |
| c.518A>G, | p.(E173G) | c.518A>G, | p.(E173G) | **increased** | 17:38241010A>G | **this report** |
| c.620G>A | p.(G207E) | c.620G>A | p.(G207E) | n.i. | 17:38243003G>A | (van Gucht et al. 2017) |
| c.632A>G | p.(D211G) | c.632A>G | p.(D211G) | decreased | 17:38243015A>G | (van Gucht et al. 2016) |
| c.641C>G | p.(A214G) | c.641C>G | p.(A214G) | n.i. | 17:38243024C>G | CinVar VCV000847657* |
| c.776T>C | p.(M259T) | c.776T>C | p.(M259T) | decreased | 17:38244547T>C | (le Maire et al. 2020) |
| c.787G>T | p.(A263S) | c.787G>T | p.(A263S) | weak decrease | 17:38244558G>T | (Demir et al. 2016) |
| c.788C>T | p.(A263V) | c.788C>T | p.(A263V) | decreased | 17:38244559C>T | (Moran et al. 2017) |
| c.817A>G | p.(T273A) | c.817A>G | p.(T273A) | decreased | 17:38244588A>G | (le Maire et al. 2020) |
| c.821T>C | p.(L274P) | c.821T>C | p.(L274P) | decreased | 17:38244592T>C | (Moran et al. 2017) |
| c.871G>A | p.(G291S) | c.871G>A | p.(G291S) | n.i. | 17:38244642G>A | (Korkmaz et al. 2019) |
| c.1053C>G | p.(H351Q) | c.1053C>G | p.(H351Q) | decreased | 17:38245529C>G | (Kalikiri et al. 2017) |
| c.1075A>T | p.(N359Y) | c.1075A>T | p.(N359Y) | decreased | 17:38245551A>T | (Espiard et al. 2015) |
| c.1099C>A | p.(L367M) | c.1099C>A | p.(L367M) | n.i. | 17:38245575C>A | (Kalikiri et al. 2017) |
| c.1110G>C | p.(K370N) | c.1110G>C | p.(L367M) | n.i. | 17:38245586G>C | CinVar VCV000029914* |
| c.1138_1141delTGCC | p.(C380Tfs*8) | intron 9 | | decreased | 17:38245614_38245617delTGCC | (Demir et al. 2016) |
| c.1139_1139delG | p.(C380Sfs9*) | intron 9 | | n.i. | 17:38245615_38245615delG | (Furman et al. 2020) |
| c.1144_1144delG | p.(A382Pfs*7) | intron 9 | | decreased | 17:38245620_38245620delG | (Moran et al. 2013) |
| c.1144G>C | p.(A382P) | intron 9 | | n.i. | 17:38245620G>C | (Kalikiri et al. 2017) |
| c.1150C>T | p.(R384C) | intron 9 | | n.i. | 17:38245626C>T | (Yuen et al. 2015) |

| | | | | | | |
|---|---|---|---|---|---|---|
| c.1151G>A | p.(R384H) | intron 9 | | decreased | 17:38245627G>A | (Demir et al. 2016) |
| c.1176C>A | p.(C392*) | intron 9 | | n.i. | 17:38245652C>A | (Tylki-Szymańska et al. 2015) |
| c.1183G>T | p.(E395*) | intron 9 | | n.i. | 17:38245659G>T | (Sun et al. 2019) |
| c.1193C>G | p.(P398R | intron 9 | | n.i. | 17:38245669C>G | (Tylki-Szymańska et al. 2015) |
| c.1190_1191insT | p.(L400Tfs*7) | intron 9 | | decreased | 17:38245666_38245667insT | (van Mullem et al. 2012, 2013) |
| c.1202T>C | p.(F401S) | Intron 9 | | weakly decreased | 17:38245678T>C | (Kalikiri et al. 2017) |
| c.1207G>T | p.(E403*) | intron 9 | | decreased | 17:38245683G>T | (Bochukova et al. 2012) |
| c.1207G>A | p.(E403K) | intron 9 | | n.i. | 17:38245683G>A | (Tylki-Szymańska et al. 2015) |
| c.1213T>C | p.(F405L) | intron 9 | | n.i. | 17:38245689T>C | (Kalikiri et al. 2017) |

## Supplemental references


Bochukova E, Schoenmakers N, Agostini M, et al (2012) A mutation in the thyroid hormone receptor alpha gene. N Engl J Med 366:243–249.

Demir K, van Gucht ALM, Büyükinan M, et al (2016) Diverse Genotypes and Phenotypes of Three Novel Thyroid Hormone Receptor-α Mutations. J Clin Endocrinol Metab 101:2945–2954.

Espiard S, Savagner F, Flamant F, et al (2015) A Novel Mutation in THRA Gene Associated With an Atypical Phenotype of Resistance to Thyroid Hormone. J Clin Endocrinol Metab 100:2841–2848.

Furman AE, Dumitrescu AM, Refetoff S, Weiss RE (2020) Early diagnosis and treatment of an infant with a novel THRA gene (pC380SfsX9) mutation. Thyroid. https://doi.org/10.1089/thy.2020.0695

Kalikiri MK, Mamidala MP, Rao AN, Rajesh V (2017) Analysis and functional characterization of sequence variations in ligand binding domain of thyroid hormone receptors in autism spectrum disorder (ASD) patients. Autism Research 10:1919–1928.

Korkmaz O, Ozen S, Ozdemir TR, et al (2019) A novel thyroid hormone receptor alpha gene mutation, clinic characteristics, and follow-up findings in a patient with thyroid hormone resistance. Hormones 18:223–227.

le Maire A, Bouhours-Nouet N, Soamalala J, et al (2020) Two Novel Cases of Resistance to Thyroid Hormone Due to THRA Mutation. Thyroid 30:1217–1221.

Moran C, Agostini M, McGowan A, et al (2017) Contrasting Phenotypes in Resistance to Thyroid Hormone Alpha Correlate with Divergent Properties of Thyroid Hormone Receptor α1 Mutant Proteins. Thyroid 27:973–982.

Moran C, Schoenmakers N, Agostini M, et al (2013) An Adult Female With Resistance to Thyroid Hormone Mediated by Defective Thyroid Hormone Receptor α. J Clin Endocrinol Metab 98:4254–4261.

Sun H, Wu H, Xie R, et al (2019) New Case of Thyroid Hormone Resistance α Caused by a Mutation of THRA/TRα1. J Endocr Soc 3:665–669.

Tylki-Szymańska A, Acuna-Hidalgo R, Krajewska-Walasek M, et al (2015) Thyroid hormone resistance syndrome due to mutations in the thyroid hormone receptor α gene (THRA). Journal of Medical Genetics 52:312–316.

van Gucht ALM, Meima ME, Zwaveling-Soonawala N, et al (2016) Resistance to Thyroid Hormone Alpha in an 18-Month-Old Girl: Clinical, Therapeutic, and Molecular Characteristics. Thyroid 26:338–346.

van Gucht ALM, Moran C, Meima ME, et al (2017) Resistance to Thyroid Hormone due to Heterozygous Mutations in Thyroid Hormone Receptor Alpha. Curr Top Dev Biol 125:337–355.

van Mullem A, van Heerebeek R, Chrysis D, et al (2012) Clinical Phenotype and Mutant TRα1. New England Journal of Medicine 366:1451–1453.

van Mullem AA, Chrysis D, Eythimiadou A, et al (2013) Clinical Phenotype of a New Type of Thyroid Hormone Resistance Caused by a Mutation of the TRα1 Receptor: Consequences of LT4 Treatment. J Clin Endocrinol Metab 98:3029–3038.

Yuen RKC, Thiruvahindrapuram B, Merico D, et al (2015) Whole-genome sequencing of quartet families with autism spectrum disorder. Nature Medicine 21:185–191.
